# Imaging of Patients with Complex Hemodialysis Arterio-Venous Fistulas using Time-Resolved Dynamic CT Angiography: Comparison with Duplex Ultrasound

**DOI:** 10.1038/s41598-017-12902-6

**Published:** 2017-10-02

**Authors:** Mathias Meyer, Nicole Geiger, Urs Benck, Daniela Rose, Sonja Sudarski, Melissa M. Ong, Stefan O. Schoenberg, Thomas Henzler

**Affiliations:** 1 Institute of Clinical Radiology and Nuclear Medicine, University Medical Center Mannheim, Medical Faculty Mannheim – Heidelberg University, Mannheim, Germany; 2 Department of Surgery, University Medical Center Mannheim, Medical Faculty Mannheim – Heidelberg University, Mannheim, Germany; 3 5th Department of Medicine, University Medical Center Mannheim, Medical Faculty Mannheim – Heidelberg University, Mannheim, Germany

## Abstract

To evaluate the feasibility and potential on therapy management of time-resolved dynamic computed tomography angiography (dCTA) in patients with forearm arterio-venous fistula (AVF)/arterio-venous grafts (AVG). Thirty-five patients with complex failing forearm AVF/AVGs were examined with ultrasound and a dCTA protocol. Diagnosis and therapy management was evaluated versus duplex ultrasound (DUS) in three different readouts: 1. all dCTA datasets; 2. one arterial phase of the dCTA dataset; 3. one arterial and one venous dataset out of the dCTA dataset. All reads were performed >30 days apart from each other. Using all data of the dCTA examination, 20 patients were classified as having a stenosis >50%, 12 high-shunt flow, 11 partial thrombosis, 5 venous aneurysms and 5 complete thrombosis of their AVF/AVG grafts. This lead to 13 additional pathologic findings not visible on DUS and reclassification as normal in one patient with suspected AVF stenosis and complete thrombus on DUS. These additional findings lead to a direct change of therapeutic management in 8 patients. Compared to readout 1 (53 pathologies), readout number 2 and 3 revealed only 33 and 41 pathologies, respectively. dCTA provides additional information, improving diagnostic confidence and leading to changes in therapy management when compared to DUS alone.

## Introduction

Patients with end-stage renal disease depend on a well functioning arterio-venous fistula (AVF) or arterio-venous grafts (AVG) for hemodialysis treatment. In current clinical practice symptomatic AVF imaging is the domain of duplex ultrasound (DUS) as recommended by guidelines^1^.

However, the technique has its disadvantage. Due to high inter-reader variability and relatively restricted field of view, image findings may be difficult to demonstrate to cardiovascular surgeons and/or interventional radiologists for definitive therapy planning^2,3^. In particular there is only a limited visual 3D anatomic road map for pre-operative surgical evaluation^4,5^. Thus, a more vivid imaging technique showing anatomical details to a greater extent with less inter-reader variability would be desirable.

Very recently, dynamic low kilovolt (kV) imaging in combination with a large z-axis coverage of up to 800 mm for time-resolved dynamic CT angiography (dCTA) has become clinically available^6^. Dynamic imaging could offer a solution, especially in the assessment of hemodynamic inadequate working complex AVF or AVG, as the arterial and venous situation of the extremity can be evaluated in one examination. However, in recent years high radiation dose levels associated with dCTA have been critically discussed since only high tube voltage imaging mainly performed with 100 kVp was routinely available^7^.

Recently introduced scanner generations allow for routine low kV imaging down to 70 kVp due to an increased maximum tube current output of up to 1300 mAs. Low kVp imaging decreases radiation exposure but further improves vascular attenuation for CTA with beneficial effects on image quality, as the effective energy of the x-ray beam in the range of maximum absorption moves closer to the k-edge of iodine (33 keV)^8^.

One further benefit of low kV dCTA imaging is the reduced required amount of contrast media for an examination, especially important in patients with end-stage renal disease but residual renal function^8^.

The purpose of this feasibility study was to clinically evaluate a dCTA low kVp imaging protocol regarding diagnostic accuracy compared to DUS for the evaluation of patients with complex AVF/AVG dysfunction and its potential influence on therapy management.

## Materials and Methods

### Patients

Our standard approach in AVF/AVG patients with inconclusive findings on DUS is a triple phase CT protocol. A recent study of our institute was able to demonstrate, that the radiation dose is similar between a dCTA protocol and a routine triple phase CT protocol on a none high-end CT scanner system^9^. Moreover, this study was designed as a proof of concept feasibility study. Therefore, this prospective single-center study was approved by the institutional review board (Clinic Ethics Committee II of Mannheim University Clinic) and complies both with the Declaration of Helsinki and the Health Insurance Portability and Accountability Act (HIPAA). The institutional review board did not demand the need for a further radiation protection agency approval. Informed consent was obtained from all participating patients.

From September 2013 to July 2015, 35 consecutive patients with AVF or AVG suspected of having a failing AVF or AVG were enrolled in this study. Detailed patient data is summarized in Table 1. Preoperative DUS and dCTA results were then compared with the gold standard, defined as surgery including intraoperative DUS with the exception of four patients were only a tight clinical follow-up with DUS was performed. These four patients had either no pathology (1 patient) on dCTA or only high-flow shunts with no consecutive ischemic lower arm disease (3 patients).

**Table 1:** Imaging protocols depending on patient position and renal function.

	Protocol A n = 5	Protocol B n = 8	Protocol C n = 10	Protocol D n = 12
Patients renal function	Partial renal function	Partial renal function	Anuria	Anuria
Central drain evaluable	no	yes	no	yes
Extremity positioning	Extremity over head	Extremity 90° angulated beside the thorax	Extremity over head	Extremity 90° angulated beside the thorax
Patient position	Abdominal position	Supine position	Abdominal position	Supine position
*Scan parameter*				
Tube voltage [kV]	70 kV	80 kV	70 kV	80 kV
Effective current-time product [mAs]	150/180 mAs	180 mAs	150/180 mAs	180 mAs
Dose length product [mGy*cm]	716 ± 58	1246 ± 76	734 ± 63	1223 ± 82
Effectiv radiation dose [mSv]	0.36 ± 0.03	17.4 ± 1.1	0.37 ± 0.03	17.1 ± 1.2
Scan range [mm]	265–340	454–630	265–340	454–630
*Scan cycles*				
Temporal resolution 2 sec	6	—	6	—
Temporal resolution 4 sec	4	—	4	—
Temporal resolution 6 sec	2		2	
Temporal resolution 2.5 sec	—	6	—	6
Temporal resolution 5 sec	—	4	—	4
Temporal resolution 7.5 sec	—	2		2
Total scan time [sec]	40 sec	50 sec	40 sec	50 sec
*Contrast media (iomeprol 400 mg iodine/ml)*				
Amount	18 g/45 ml	18 g/45 ml	32 g/80 ml	32 g/80 ml
Flow rate [ml/sec]	5 ml/sec	5 ml/sec	5 ml/sec	5 ml/sec

Notes **–** kV = kilo voltage.

### Duplex ultrasound evaluation

All patients were evaluated by B-mode and color doppler/duplex imaging for simultaneous visualization of vessel anatomy and blood supply before dialysis sessions and prior to the dCTA (mean time 6 days [range 3–12 days]). DUS evaluations were carried out by means of a color flow scanner (Hitachi, Model EUB-7500 HI-Vision CV LCD) with a 2-D linear phased array probe at high frequencies ranging from 5 to 10 MHz, as appropriate. Vascular structures of all fistulas (AVF and AVG) were assessed longitudinally and transversely for both arterial and venous districts. Starting with the subclavian arteries, examinations proceeded to the brachial arteries, anastomoses, perforator veins as well as forearm and upper arm cephalic, basilic, brachial veins and terminating at the central subclavian veins. Assessments comprised internal vessel diameters, wall thickness and irregularities, vessel courses, any steno-occlusive lesions, focal color aliasing, peak systolic velocities and blood flow rates, as required. Flow measurements were taken either in a standard place 5 cm above the elbow or aiming at an angle of insonation below 60° and were automatically calculated, as reported elsewhere^2,10^. DUS was performed and interpreted in the department of vascular surgery by two independent physicians (either vascular surgeon or a nephrologist) with >5 years of experience in AVF/AVG DUS assessments.

### CT Technique

**Figure 1:**
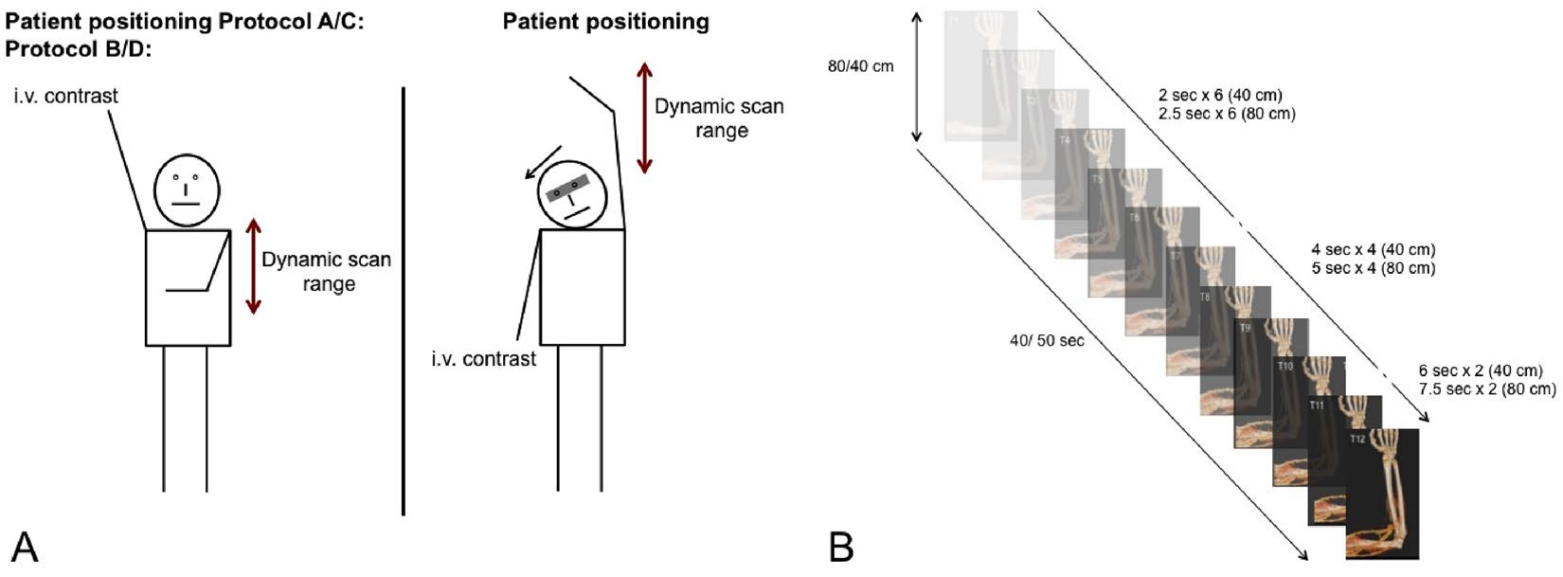
Schematic overview of the time-resolved dynamic CT angiography scan protocols. Image (**A**) displays the four different scanning positions with the differing scanning ranges. Depending on the scan range, the temporal resolution changes per scan as displayed in image (**B**).

All CT examinations were performed on a third generation dual-source CT (DSCT) system (SOMATOM Force, Siemens Healthineers, Forchheim, Germany). A total of 12 dynamic phases were acquired in each patient resulting in a scan time of 40–50 sec (for details see Table 1 and Fig. 1). The imaging protocol was adjusted depending on the patients’ renal function and the necessity of the evaluation of the central venous drainage. All patients received hemodialysis immediately after the dCTA examination via a central dialysis catheter, if their AVF/AVG was completely malfunctioning. The glomerular filtration rate was determined within 48 h prior to the dCTA examination and 24 h, 48 h and 72 h after the dCTA examination.

I. Scan protocol for patients with impaired renal function and residual renal function (compare Fig. 1)
  - If there was a necessity of evaluating the patients’ central drainage, patients’ AVF/AVG extremity was angulated by 90° besides the thorax in supine position (compare Fig. 1). Patients received a total of 18 g iodine for the contrast enhanced scan (Protocol A).
  - If there was no necessity of evaluating the central drainage patients, patients’ AVF/AVG extremity was evaluated above the head (compare Fig. 1). Patients received a total of 18 g iodine for the contrast enhanced scan (Protocol B).
II. Scan protocol for patients with impaired renal function and anuria:

- If there was a necessity of evaluating the patients’ central drainage, patients’ AVF/AVG extremity was angulated by 90° besides the thorax in supine position. Patients received a total of 32 g iodine for the contrast enhanced scan (Protocol C).
- If there was no necessity of evaluating the patients’ central drainage, patients’ AVF extremity was evaluated above the head. Patients received a total of 32 g iodine for the contrast enhanced scan (Protocol D).

Table 1 summarizes the scan parameters of all four imaging protocols.

### Image analysis

For image analysis all dCTA data sets were transferred to a dedicated offline workstation (Syngo.Via VA 30, Siemens Healthineers, Forchheim, Germany).

The readings were performed by two experienced radiologists (with 4 years and 8 years in CTA) and an experienced vascular surgeon specialized in AVF/AVG surgery (with >10 years) in consensus. The dCTA datasets were displayed using a dynamic visualization in freely angulated multiplanar reconstructions that allowed variable maximum intensity reformation, as well as various dynamic volume rendering reconstruction technique (VRT) reconstructions. Each dCTA dataset included 12 dynamic phases. These could be advanced and stopped at any time point. Furthermore, dCTA datasets in transverse sections, multi-planar reformations, and 1.5mm-thick maximum intensity projections were used for evaluation. For dCTA, the following subjective and objective evaluations were performed for the afferent and efferent vessels of the AVF/ AVG itself. Further, the assessment of arterial or venous stenoses was dichotomuos performed: 1, no significant stenosis (<50%) or 2, significant stenosis (>50%). A subjective evaluation was performed in order to identify high-flow shunts. These were defined as a very early filling of the efferent venous vessel and a delayed (<2 sec) filling of the lower upper extremity arterial vessels.

In total, three different CT readouts were performed and compared to the DUS findings:

1. After DUS was performed patients received their dCTA scan within 12 days. In this first read images were evaluated using all datasets by the above-mentioned two radiologists and vascular surgeon.
2. In a second reading at least 30 days later, the images were assessed in consensus by the same radiologists and vascular surgeon in a blinded and randomized fashion for the vascular abnormalities using a single dataset with a fixed delay after contrast media injection of 18 sec (single phase read).
3. In a third evaluation, again at least 30 days later as read number 2, images were interpreted using the best arterial dataset and the best venous dataset (dual phase read). Again, the same readers assessed the images in a blinded and randomized fashion. Best arterial/venous phase was defined with the highest HU in the artery and vein.

CT attenuation of tissues (in HU), image noise (standard deviation of CT attenuation values) and contrast-to-noise-ratio (CNR) were evaluated by one observer (M.M.) with 4 years of experience in CTA. To determine the attenuation values and the CNR, standardized 0.2 cm^2^ regions of interest were placed in different vessel regions using a dedicated assessment tool (Syngo.Via VA 30, Siemens Healthineers, Forchheim, Germany):

- Protocol A/C:
  - Artery: afferent artery 15 cm proximal of the anastomosis
  - Vein: efferent vein 20 cm after the anastomosis
  - Distal artery: 10 cm distal of the anastomosis
- Protocol B/D:
  - Artery: afferent artery 15 cm proximal of the anastomosis
  - Vein: efferent vein 15 cm after the anastomosis
  - Distal artery: 10 cm distal of the anastomosis

Subjective image quality was initially assessed independently by two observers (﻿T.H. /M.M.,8 and 4 years experience in CTA, respectively) and subsequently in a consensus reading to reconcile discrepant scores. Both observers rated overall image quality, overall image noise and motion artifacts according to a 3-point-Likert-scale (1 = excellent image quality/no noise/no motion artifacts; 2 = good image quality/some noise/slight motion; 3 = non-diagnostic/high image noise/high motion artifacts) in accordance with the criteria described for chest CT examinations in the European Guidelines on Quality Criteria for CT^11^.

### Statistical Analysis

All statistical analyses were performed using SAS 10.0 software (SAS Institute, Cary, North Carolina, USA). A p-value of 0.05 was considered to indicate statistical significance. Continuous variables were expressed as mean ± standard deviation, and categorical variables as percentages. Comparisons between the low and high contrast protocol as well as between the different findings were analyzed with two-way analysis-of-variance (ANOVA), if data was normally distributed according to the Shapiro-Wilk Test. The Kruskal-Wallis two-way analysis-of-variance was used if data was not normally distributed.

## Results

### Patient characteristics

Patients were predominantly male (63%), had a mean age of 62 years (range 39–91), and had a mean BMI of 26.8 kg/m^2^. Detailed patient characteristics are summarized in Table 2. All examinations were completed successfully without any complications (no technical or contrast timing error). There was no significant decrease in glomerular filtration rate within the 72 h post dCTA examination time interval (compare Table 2; p > 0.05).

**Table 2:** Demographics of all patients included in this study.

Characteristic	Values	
Patients	35	
Male patients	20	
Age (years)	62 ± 18	
BMI (kg/m^2^)	26.8 ± 3.1	
Mean age of fistula (months)	17 ± 9	
AV fistula	27	
AV graft	8	
Type of AV fistulas/grafts		
Forearm fistula/graft	24	
Upper arm fistula/graft	11	
Glomerular filtration rate [ml/min]		*p*-Value (vs prior CT)
Prior to CT	6.1 ± 2,1	—
24 h post CT	6.3 ± 2,0	0.7961
48 h post CT	6.7 ± 1,6	0.4452
72 h post CT	6.2 ± 1,7	0.8417

Note – BMI = body-mass-index; AV = arterio-venous; CT = computed tomography.

### Image quality of time resolved dynamic CTA datasets

All 35 patient examinations were rated as diagnostic with an overall image quality of 1 (25–75% quantiles: 1–1). Similar image noise was excellent with a median image noise of 1 (25–75% quantiles: 1–2). Significantly more patients were rated with higher image noise for the scan protocol with the angulated arm to the thorax when compared to the overhead protocols (p = 0.0151). As expected, the maximum values of the 12 phases showed significantly different mean attenuation and CNRs between the high contrast and the low contrast scan protocol (compare with Table 3; p < 0.05).

**Table 3:** Attenuation and CNR of the best phase for different vessel regions.

	Protocol A Low CM Volume	Protocol B Low CM Volume	Protocol C High CM Volume	Protocol D High CM Volume	P-value* High vs low CM Volume
*Attenuation (HU)*					
Artery	471 ± 66	435 ± 77	561 ± 69	534 ± 102	P < 0.0017
Shunt	469 ± 65	432 ± 75	557 ± 69	528 ± 103	P < 0.0023
Vein	204 ± 83	224 ± 55	345 ± 68	325 ± 91	P < 0.0001
Distal artery	380 ± 68	364 ± 61	461 ± 69	425 ± 108	P < 0.0071
*CNR*					
Artery	13 ± 3	10 ± 2	14 ± 3	14 ± 4	P < 0.0239
Shunt	13 ± 3	10 ± 2	14 ± 3	14 ± 3	P < 0.0223
Vein	5 ± 3	4 ± 2	8 ± 2	8 ± 3	P < 0.0002
Distal artery	10 ± 2	8 ± 2	11 ± 2	10 ± 4	P < 0.0468

Note – CNR = contrast-to-noise ratio; CM = contrast media.

### Time and delay of contrast enhancement

The mean delay for the best enhancement of the AVF or AVG after injection was 18 sec (range 12–25 sec). In comparison, the best venous phase after injection was 23 sec (range 15–33 sec).

The time delay of both, the initial enhancement (2.4 ± 0.8 sec vs. 3.7 ± 1.1 sec; p < 0.05) and the enhancement peak (2.1 ± 0.6 sec vs. 3.9 ± 0.9 sec; p < 0.05) from the artery to the distal artery were significantly longer for patients with a high-flow shunt when compared to the rest. Similar patients with complete occluded proximal shunt vein showed a significantly time delay from the artery to the vein, again for both the initial enhancement (2.9 ± 2.0 sec vs. 6.1 ± 2.2; p < 0.05) and the enhancement peak (2.7 ± 1.9 sec vs. 5.5 ± 2.1 sec; p < 0.05).

### Diagnostic accuracy of the four different readouts

In the initial DUS read a significant stenosis was diagnosed in 19 patients, complete thrombosis and partial thrombosis in 6 patients each, a high-flow shunt in 6 and a venous aneurysm in 5 patients. This lead to a sum of 42 abnormalities in 35 patients.

**Figure 2:**
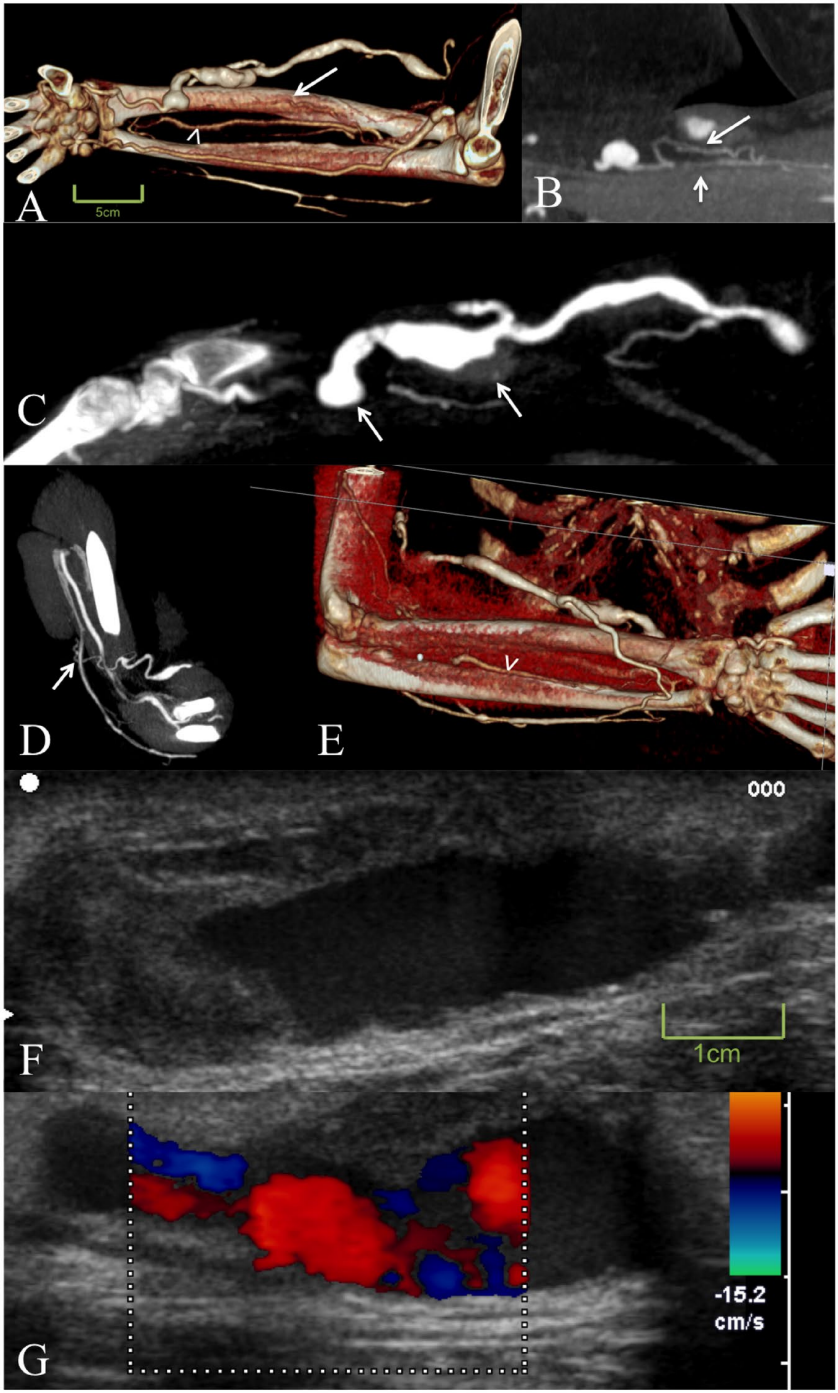
Time-resolved dynamic CT angiography of a 57-year-old female patient with a complex failing arterio-venous forearm fistula. Image (**A**) shows only marginal contrast of the afferent radial artery with a stenosis 2 cm proximal to the arterio-venous forearm fistula and a collateral artery as a sign for a long term stenosis (arrows in **B**). Image (**A**) also demonstrates a very strong anterior interosseous artery (arrow head in **A** and **E**) also indicating a chronic stenosis. Further, the study revealed two venous aneurysms with a wall adherent thrombus directly located after the fistula (arrows in **C**). The venous return revealed only drainage over the deep perforator venous system without a connection to the cephalic vein (arrow in **D**). The main venous return is depicted in image (**E**) via a dilated superficial vein to the basilica vein. Duplex ultrasound images in F and G demonstrate the partially thrombotic aneurysm but do not reveal the stenosis proximal to the fistula, as well as the insufficient venous backflow to the cephalic vein.

**Figure 3:**
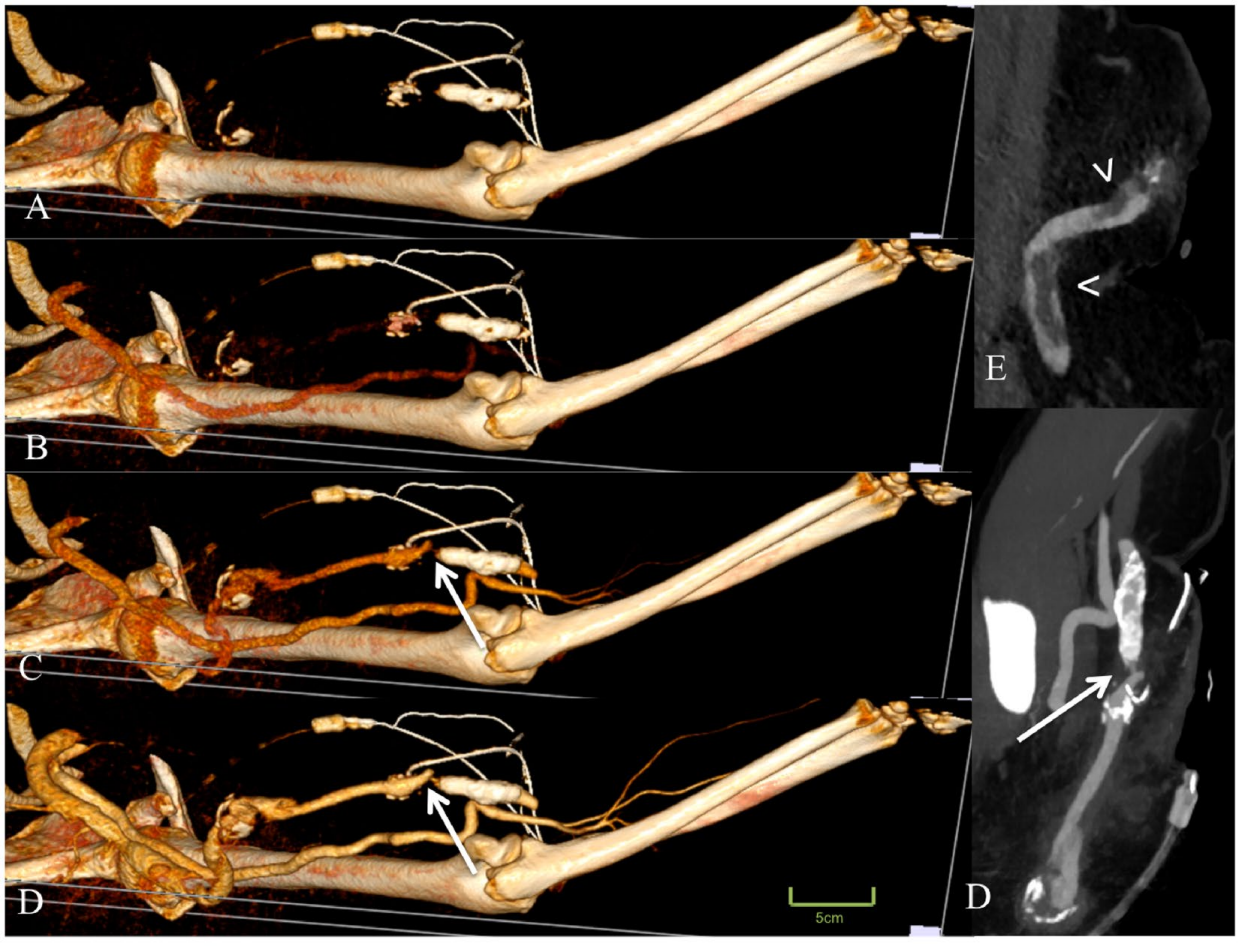
Displayed is a 50-year old female patient with a complex arterio-venous graft and status post multiple revisions, with a new dysfunction of her arterio-venous graft. Time-resolved dynamic CT angiography displayed a high-grade stenosis of the efferent vessel (arrows in **C** and **D**). In addition, a thrombus could be detected in the subclavian vein (arrow heads in **E**).

**Figure 4:**
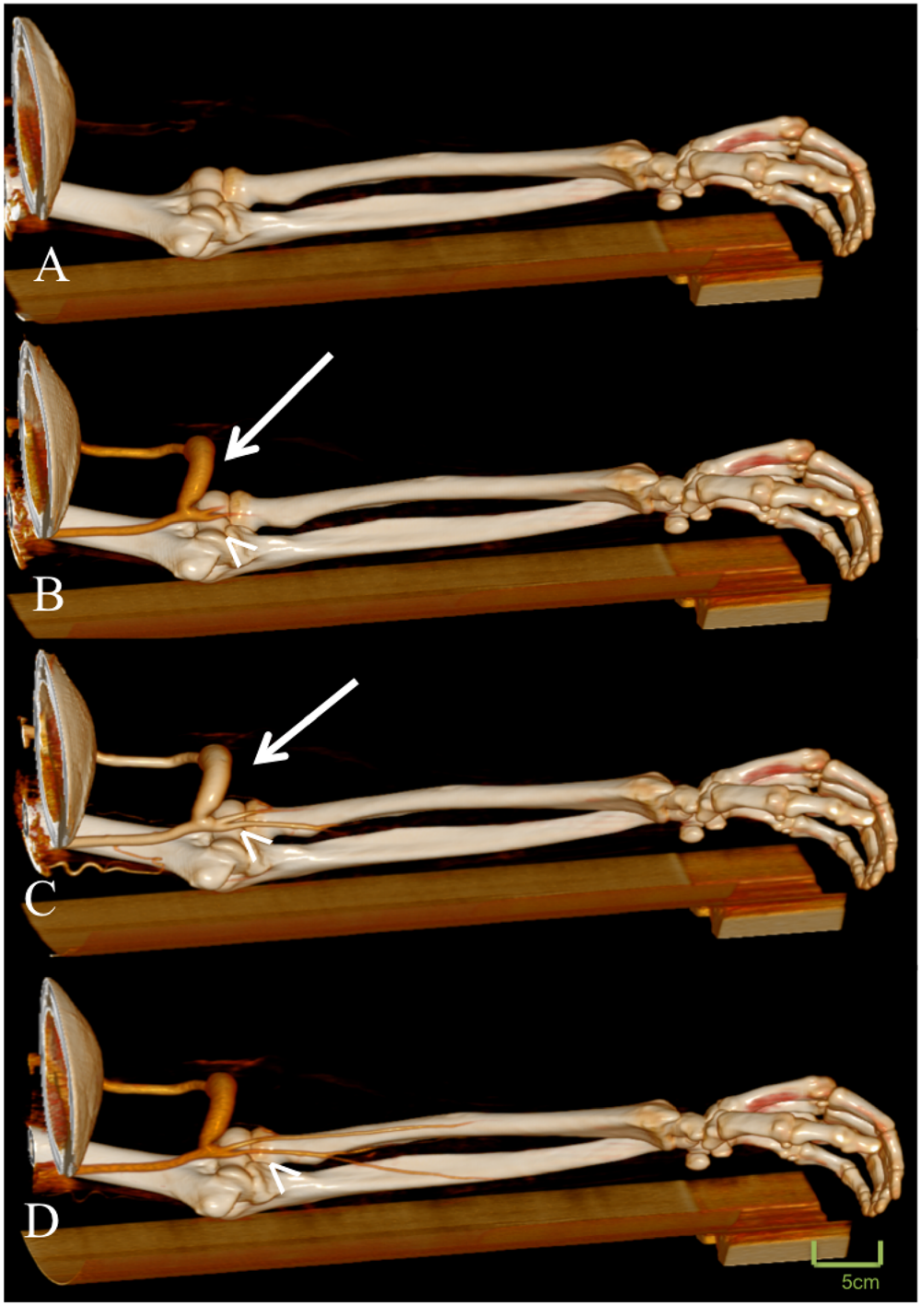
Displayed is a 48-year old male patient with new onset of arm pain. The patient had revision surgery due to a failing arterio-venous failure two weeks prior to the examination. Time-resolved dynamic CT angiography displayed an early filling of the efferent vein and a delayed filling of the brachial artery (arrow head) indicating a high-flow shunt (see also supplementary video 1).

The dCTA using all phases (read 1) revealed a significant stenosis in 20 patients, complete thrombosis in 5, partial thrombosis in 11, a high-flow shunt in 12, venous aneurysm in 5 and no abnormalities in 1 patient (Figs 2–4 and movie 1, 2). Six high-flow shunts as seen on dCTA had been missed by DUS. All of these patients had undergone multiple AVF/AVG revisions, with the vessel anatomy being unclear, and the AVF/AVG had not been used after the last revision. The initial DUS was scheduled to evaluate the AVF/AVG for usage. After dCTA revealed the underlying complex vessel anatomy all high-flow shunts could be located and confirmed by DUS. Five partially thrombosis were additionally detected by dCTA. In two patients dCTA revealed high-grade stenosis. In one patient DUS suspected a complete thrombosis and a high-grade stenosis, dCTA revealed no abnormalities at all. All partial thrombosis detected on DUS could be seen and confirmed by dCTA. Of the 6 complete thrombosis detected by DUS only 2 could be seen and confirmed by dCTA. The other 3 were reclassified as partial thrombosis. However, 4 additional complete thrombosis were missed on DUS, that were present on dCTA.

These additional findings led to a direct therapy change in 8 patients. In particular, in the 2 patients were significant stenosis were missed, this lead to surgical interventions (one new AVG and one new AVF). The patient with no abnormalities on dCTA, was only closely followed by DUS with no intervention being performed. In 3 patients with missed high-flow shunts on DUS, the findings by dCTA lead to a revision of the shunt. Additionally 2 patients with missed complete thrombosis on DUS and detected significant AVF/AVG stenosis, which were initially scheduled for endovascular treatment underwent direct surgery and received new AVFs.

In comparison, only 33 abnormalities were seen after assessing the single phase interpretation read (read 2), mainly missing the thrombosis and the high flow shunt. This would have led to a different therapy in 16 patients when compared to the all phase dCTA read (read 1).

**Figure 5:**
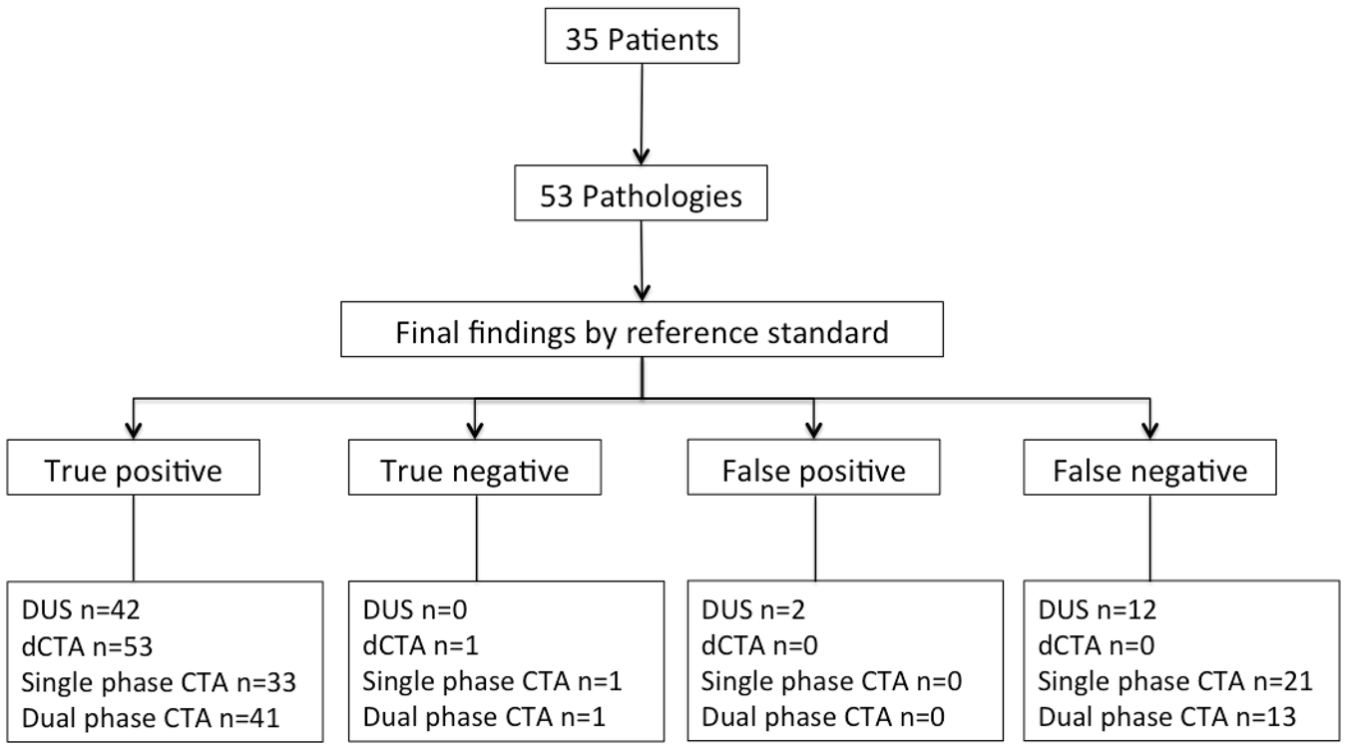
Patient flow chart displaying the pathology findings of each modality in comparison to the gold standard (DUS = duplex ultrasound; dCTA = time-resolved dynamic computed tomography angiography).

When considering the findings of the dual-phase interpretation read (read 3) 41 abnormalities were found. All high flow shunts and one partial thrombosis was also missed. This would have led to a different therapy in 10 patients when compared to the all phase dCTA read (read 1). Detailed results of each reading are summarized in Table 4 and in Fig. 5.

**Table 4:** Detection rate of vascular pathologies using different phases, duplex ultrasound in comparison to the dCTA findings.

	dCTA Interpretation	Single phase read	Dual-phase read	Duplex ultrasound
Stenosis >50%	20	20	20	19
Complete thrombosis	5	3	5	6
Partial thrombosis	11	4	10	6
High-flow shunt	12	0	0	6
Venous aneurysm	5	5	5	5
No abnormalities	1	1	1	0

Note – dCTA = time-resolved dynamic CT angiographie; single phase = fixed delay after contras media injection of 18 sec; dual-phase = best arterial phase and best venous phase.

## Discussion

Although DUS has its well know restrictions, it is available around the clock, inexpensive, and a noninvasive method for evaluating symptomatic AVF or AVGs. Therefore, it is recommended by recent guidelines^1^. Especially evaluating stenoses in the venous outflow tract and in particular the subclavian vein can be challenging. This is particularly because of close anatomic relationship to the clavicle and its deep location^12^. Nevertheless, the assessment of the central venous outflow is essential, as the subclavian vein frequently shows venous stenosis in patients on dialysis^13,14^.

The first standard CTA on evaluating AVF and AVG dysfunction in hemodialysis patients dates back 16 years. Although these early studies demonstrated a high sensitivity, they were limited to small patient populations and restricted scan coverage with an inferior spatial resolution, not able to cover the entire AVF/AVG^15,16^. Later studies using 4–64 slice scanner demonstrated a sensitivity ranging between 90.2–98.7% and specificity between 92.2–97.5%^17–20^. However, these studies only evaluated the presence or absence of stenosis in an AVF/AVG. Since AVF/AVG are more complex there are a number of reasons for failure besides significant stenoses. Within this context, dCTA imaging could offer a solution, especially in the assessment of hemodynamically inadequate working complex AVF or AVG, as the arterial and venous situation of the extremity can be evaluated in one examination.

In our study, 16 patients had either partially or complete thrombosis of the venous outflow tract, of which five were not detected by DUS. This emphasizes the importance of a non-invasive evaluation tool, which can assess the deep venous system, while providing both overarching anatomic and functional analysis. In the one patient where DUS has missed the significant stenosis, the patient had undergone multiple diagnostics, including interventional digital subtraction angiography (DSA) sessions, prior to the dCTA study. dCTA, as well as single and dual-phase CTA was able to reveal a severe kink of the proximal part of the radial artery as cause for the failure. This kinking was missed by DSA and DUS due to vessel overlap, which is a well-known drawback of these techniques^5^. Based on the dCTA images a surgical graft interposition of the kinked artery led to flow recovery. Further, from a vascular surgeon standpoint DUS only provides partial information of the AVF or AVG and the surrounding vessels as it has a limited field of view and tissue penetration. In contrast, dCTA datasets of the entire upper extremity or the entire forearm have the advantage of supplying a vascular surgeon with the anatomic vessel information. Additionally, functional information in delayed enhancement of occluded vessels and the clinical relevance of these stenoses can be provided.

Although the contrast media amount was decreased to 18 g in our low contrast media protocol, we found sufficient high attenuation values of the upper extremities vessels. This is significantly less compared to our standard CTA protocol and those of reported studies using contrast media amounts of 30–32 g or more^5^. Using a low contrast media protocol is especially important in order to preserve renal function in those patients with an AVF or AVG and partial renal function.

Our study protocol consisted of 12 low-dose scan phases with a temporal resolution of one scan every 2–5 seconds and showed sufficient confidence for detecting and classifying failing AVF or AVGs while also offering dynamic information. Although there is an increased number of CT scans with a dCTA protocol, the overall radiation exposure can be maintained, as the tube voltage has been decreased to 70/80 kVp. One further benefit of CT scans at lower tube voltage (70/80 kVp), is the fact of an improved intravascular attenuation^21^.

When scanning patients with their hands above their heads and a scan range of 265–340 mm the mean effective radiation dose was 0.36 mSv. With an increased scan range of 454 mm and evaluating the central venous outflow tract the radiation dose was in the range of a bi- to triphasic routine chest protocol, without using modern radiation dose reduction algorithms^22,23^. When comparing the radiation dose to modern triphasic CT protocol with modern reduction techniques such as automated tube current and tube voltage modulation as well as iterative reconstruction the radiation dose levels were 29% higher (approximately 12.2 mSv vs 17.4 mSv)^24^.

However, alternative to dCTA DSA with complete access, including in- and outflow, in combination with DUS can be considered, as this may result in similar diagnostic accuracy for identifying pathologies in complex failing AVF/AVG^12^. Next to the opportunity of a direct intervention, a percutaneous angiography without intervention maybe associated with a lower radiation and contrast media exposure (e.g. 2.6 mSv and 3.5 g)^12,25^. On should however, outweigh the risks of complications such as access rupture or access thrombosis or access hematoma in each patient separately.

Our study may have some limitations: Although dCTA may have shown a better performance than DUS, we do not advocate the routine use of dCTA for the evaluation of AVFs. First of all, when evaluation the central outflow track, dCTA is associated with an increased radiation dose. In our study the maximum radiation exposure was 19 mSv. The relative cancer risk for a radiation doses of <100 mSv lies between 1.001 and 1.04 in this patient cohort^26^. As patients with end stage renal insufficiency have high 5-year mortality rate (attributed 5-year survival rate of patients with AVF/AVGs is 35.8%^27^), we believe that the associated risk of an increased radiation exposure is outweighed by the diagnostic benefits and changes in therapy for this particular patient cohort. Secondly, dCTA always requires a venous puncture in order to inject the contrast agent. This is in contrast the doctrine of venous preservation, where punctures of peripheral veins should kept to a minimum, as recommended by the Dialysis Outcome Quality Initiative guidelines^28^. Third, our study population is somehow preselected only consisting of complex AVF/AVG failures, which may have led to a selection bias at the cost of DUS. Fourthly, in our study the gold standard was defined as surgery including intraoperative DUS, which was performed in 89% of the patients. In the remaining 11% of patients, the risk of surgical evaluation would have not been justified. Therefore our study leaks a true none surgical gold standard in these patients. DSA is considered the gold standard for the evaluation of vessel patency. However, CTA has been proven to be an accurate alternative in peripheral artery disease with sensitivity and specificity >90%^29^. Further, we found a high number of high-flow shunts in these patients as the only underlying pathology. Identification and interpretation of these high-flow shunts on DSA can be challenging. Finally, our study results may have been influenced by the fact that each DUS performed was only evaluated by two readers, whereas the dCTA studies were evaluated by three readers and the all phase dCTA study in an interdisciplinary consensus. This may have led to an underestimation of the DUS results. Although DUS results are very much examiner dependent, a triple reading is not feasible in daily routine. Despite these limitations dCTA has the potential to add additional information in complex or unclear cases of failing AVF or AVGs and guide surgeons where DUS may fail as an add-on examination.

In conclusion, this preliminary study demonstrates the potential of dCTA in patients with complex AVF or AVG failing. Time-resolved dynamic CTA proved to be a reliable and reproducible tool for the assessment of patients with complex failing arterio-venous fistulas where duplex ultrasound may have its limitations. However, as dCTA is associated with an increased radiation dose, it should be considered as an add-on assessment tool in complex cases.

## Electronic supplementary material

Supplementary information

supplementary video 1

supplementary video 2
