# Supplementary material for: Imaging of Patients with Complex Hemodialysis Arterio-Venous Fistulas using Time-Resolved Dynamic CT Angiography: Comparison with Duplex Ultrasound
Source: Sci Rep. 2017 Oct 2;7:12563. doi: 10.1038/s41598-017-12902-6 (PMC5624919; doi:10.1038/s41598-017-12902-6)
Supplement: Supplementary file 1 — Supplementary information [file 41598_2017_12902_MOESM1_ESM.doc]

***Imaging of Patients with Complex Hemodialysis Arterio-Venous Fistulas using Time-Resolved Dynamic CT Angiography: Comparison with Duplex Ultrasound***

Mathias Meyer, MD^1^; Nicole Geiger, MD^2^; Urs Benck, MD^3^; Daniela Rose, MD ^3^; Sonja Sudarski, MD^1^; Melissa M. Ong, MD^1^; Stefan O. Schoenberg, MD^1^; Thomas Henzler, MD^1^

*General information and ethic approval*

Our standard approach in AVF/AVG patients with inconclusive findings on DUS is a triple phase CT protocol. A recent study of our institute was able to demonstrate, that the radiation dose is similar between a dCTA protocol and a routine triple phase CT protocol on a none high-end CT scanner system ^1^. Moreover, this study was designed as a proof of concept feasibility study. Therefore, this protocol was approved by our local ethics committee (Clinic Ethics Committee II of Mannheim University Clinic), which did not demand the need for a further radiation protection agency approval.

*CT Technique:*

The DSCT was used in single-source mode and a 0.32 sec gantry rotation time and a 1.5 x 48 x 1.2 mm collimation. All patients were examined using either a tube voltage of 70kV or 80kV and a fixed effective tube-current product of either 150 mAs or 180 mAs depending on the protocol chosen. For those patients undergoing protocol B and D, an eye lens protection shield (CT-Eye-ProteX, SOMATEX Medical Technologie GmbH, Teltow, Gerany) was used in order to minimize the eye lens organ dose. The dynamic time-resolved scan range depended on the patient size and the regions being assessed from 265 - 630 mm with a temporal resolution of 2 - 2.5 sec.

All dCTA data were reconstructed with a slice thickness of 1.5mm using an increment of 1.0 in the axial plane. Images were reconstructed with filtered back projection using a dedicated vascular kernel (Bv40).

Contrast media was injected at a flow rate of 5mL/sec through an 18-gauge intravenous antecubital catheter, followed by 50mL of saline at the same flow rate. The contrast media (iomeprol 400mg/100ml [Imeron 400, Bracco Imaging S.p.A., Milan, Italy]) was either chosen with 18 g or 32 g depending on the partial renal function. A fixed delay of 8 sec was chosen for each scan. Table 1 summarizes the scan parameters of all four imaging protocols.

*Image analysis*

The phase with the highest attenuation was determined, and corresponding Hounsfield units (HU) were measured. CNR was determined for the different vessels using the following formula:

CNR =
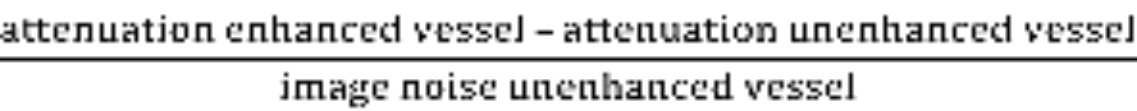


Image noise was defined as the standard deviation of the HU.

*Assessment of Radiation Dose*

For each patient, the volume CT dose index (CTDI_vol_) and dose length product (DLP) were noted. Effective doses (ED) were estimated from the DPL values according to the generic method presented in the ‘*European Guidelines for Multislice Computed Tomography*’ using a specific dose conversion coefficient for the chest of 0.014 mSv Gy^-1^ cm^-1^ and for extremities of 0.0005 mSv Gy^-1^ cm^-1^ ^2^.

*Supplementary videos*

**Movie 1** – Displayed is a 48-year old male patient with new onset of arm pain. The patient had revision surgery due to a failing arterio-venous failure two weeks prior to the examination. Time-resolved dynamic CT angiography displayed an early filling of the efferent vein and a delayed filling of the radial and ulnar artery (arrow heads) indicating a high-flow shunt

**Movie 2** – Displayed is a 79-year old male patient with new ischemic hand pain and a failing forearm AVF. Time-resolved dynamic CT angiography displayed a complete occlusion of the distal radial artery (white arrow). Further, multiple high grade stenosis are present in the ulnar artery with a complete occlusion in the distal part (red arrow). A strong interosseous artery, with retrograde filling of the distal radial artery, forms the palmar arch. Time-resolved dynamic CT angiography detected a small aneurysm in the afferent artery with a subtotal occlusion of the forearm AVF and the efferent vein (blue arrow).

1. Gawlitza, J.*, et al.* Comparison of organ-specific-radiation dose levels between 70 kVp perfusion CT and standard tri-phasic liver CT in patients with hepatocellular carcinoma using a Monte-Carlo-Simulation-based analysis platform. *European journal of radiology open* **3**, 95-99 (2016).

2. The 2007 Recommendations of the International Commission on Radiological Protection. ICRP publication 103. *Ann ICRP* **37**, 1-332 (2007).
